# Neutrophils enhance the clearance of systemic amyloid deposits in a murine amyloidoma model

**DOI:** 10.3389/fimmu.2024.1487250

**Published:** 2024-11-12

**Authors:** Trevor J. Hancock, Marina Vlasyuk, James S. Foster, Sallie Macy, Daniel C. Wooliver, Manasi Balachandran, Angela D. Williams, Emily B. Martin, Stephen J. Kennel, Eric R. Heidel, Jonathan S. Wall, Joseph W. Jackson

**Affiliations:** ^1^ Department of Medicine, University of Tennessee Graduate School of Medicine, Knoxville, TN, United States; ^2^ Department of Surgery, University of Tennessee Graduate School of Medicine, Knoxville, TN, United States

**Keywords:** AL amyloidosis, neutrophils in amyloid, neutrophil NETs, amyloid phagocytosis, amyloid resolution

## Abstract

**Introduction:**

Amyloid-specific antibodies have been shown to opsonize and enhance amyloid clearance in systemic amyloidosis mouse models. However, the immunological mechanisms by which amyloid is removed have not been clearly defined. Previous reports from preclinical *in vivo* studies suggest polymorphonuclear cells (*i.e.*, neutrophils) can affect amyloid removal. Therefore, we sought to analyze how neutrophils may contribute to the clearance of human AL amyloid extracts, using a murine amyloidoma model.

**Methods:**

Immunocompromised nude mice injected subcutaneously with patient-derived AL amyloid extract (generating a localized “amyloidoma”) were used to circumvent confounding factors contributed by the adaptive immune system and served as the model system. Two representative AL amyloid extracts were used, ALλ(CLA), which is refractory to clearance, and ALκ(TAL), which is readily cleared in mice. Neutrophil recruitment to the amyloid masses, cellular activation, and propensity to engulf amyloid were assessed.

**Results:**

Immunophenotyping of amyloidomas from animals implanted with 2 mg of either ALλ or ALκ revealed that more neutrophils were recruited to ALκ amyloid masses as compared to the ALλ material, which was generally devoid of neutrophils. *Ex vivo* analyses indicated neutrophils do not efficiently phagocytose amyloid directly. However, histological evaluation of the ALκ amyloidoma revealed the abundant presence of neutrophil extracellular traps, which were absent in the ALλ amyloidomas. Using neutrophil depletion experiments in mice, we determined that mice devoid of neutrophils cleared the human amyloid lesions less efficiently. Moreover, mice devoid of neutrophils also had significantly reduced intra-amyloid expression of inflammatory cytokines.

**Discussion:**

Neutrophils may not directly mediate amyloid clearance through phagocytosis; however, these cells can be stimulated by the amyloid and may function to facilitate phagocytosis and amyloid clearance by professional phagocytes (*e.g.*, macrophages).

## 1 Introduction

Systemic amyloidosis refers to a group of protein misfolding disorders in which precursor proteins aggregate to form amyloid fibrils (1, 2). These fibrils, along with various accessory proteins, deposit in the extracellular space of visceral organs and tissues which can compromise organ architecture and function, resulting in patient morbidity and mortality (3). Currently, ~40 precursor proteins have been identified that undergo extracellular fibrilization, of these, ~18 have been implicated in causing systemic disease (4). The remaining amyloidogenic precursor proteins typically deposit at the site of synthesis, in a single organ (*e.g.*, neuronal β-amyloid deposition in Alzheimer’s disease or type II diabetes-related amyloid deposition in the pancreas) (5).

Immunoglobulin light chain-associated (AL) amyloidosis is a major type of systemic amyloidosis with between 30,000 to 45,000 cases in the U.S. and European Union and an estimated annual incidence of ~4,500 cases per year in the U.S. (Amyloidosis Foundation) (2, 6). However, AL amyloidosis is notoriously underdiagnosed due to disease manifestations being variable and non-specific (7). For AL amyloidosis, treatment regimens generally include some combination of chemotherapy and/or immunotherapy, which is designed to inhibit plasma cell synthesis of amyloidogenic light chains (e.g., CyBorD and daratumumab). The current standard of care has prolonged patient survival, but these treatments are not designed to address the removal of preexisting protein deposits (2, 8–10), and are generally not curative. Consequently, patient prognosis remains poor.

It is well established that amyloid burden, notably in the heart and kidneys, correlates with disease outcome. Cardiac amyloid burden, determined by histological analysis or cardiovascular magnetic resonance T1 mapping, is a predictor of mortality in patients with AL amyloidosis (11, 12). Likewise, kidney function inversely correlates with renal amyloid load (13, 14). Interestingly, some AL patients who achieve complete hematological remission (*i.e.*, normalization of the involved serum free light chain concentration), have demonstrated a reduction in amyloid burden in subcutaneous abdominal fat (15), gastric mucosa (16), the liver (17–19), and hepatosplenic reduction has been observed using the evuzamitide imaging agent (20). While encouraging, endogenous amyloid resolution does not occur in all patients with AL amyloidosis, nor is the process by which amyloid regression occurs known. However, these reports suggest there are natural mechanisms that can mediate amyloid removal.

Using a murine amyloidoma model (21) we analyzed the resolution of patient-derived amyloid implanted subcutaneously into immunodeficient mice with a focus on assessing the role of neutrophils. Two representative human AL amyloid extracts, ALκ(TAL) and ALλ(CLA), which differ in their innate clearance rates when implanted in mice, were chosen for this study. Despite being compositionally similar, as determined by mass spectrometry, electron microscopy, and fibril content, the ALκ(TAL) amyloid is cleared significantly faster than ALλ(CLA) extract. The differences in amyloid clearance rates between ALκ(TAL) and ALλ(CLA) are not likely dependent on amyloid subtype (*i.e.*, ALκ or ALλ) since we have previously identified ALκ extracts that are refractory to macrophage phagocytosis while certain ALλ extracts are easily phagocytosed (22). These extracts represent prototypic examples of AL amyloid that are readily cleared, or refractory to removal, in this mouse model. In this study, we demonstrate, using immunophenotyping experiments, that mice implanted with the ALκ amyloidoma exhibit sustained intra-amyloid neutrophil recruitment beyond the initial foreign body response following amyloid implantation, while the ALλ amyloidoma only displayed initial neutrophil recruitment following amyloid implantation. We hypothesized that differences in neutrophil accumulation may contribute to the differential clearance rates of the two amyloid extracts. Antibody-mediated neutrophil depletion experiments confirmed that amyloid dissolution was impeded in mice lacking neutrophils as compared to naïve animals. These data, in conjunction with previous observations which indicated that polymorphonuclear cells (*i.e.*, neutrophils) influence amyloid resolution following antibody therapy in a murine model of localized AL amyloidoma (23), support our hypothesis that neutrophils can contribute to amyloid resolution.

## 2 Methods

### 2.1 Cell lines and animals

Murine Raw264.7 macrophages (ATCC) were grown in DMEM F-12 (Cytiva, Marlborough, MA) supplemented with 1% penicillin/streptomycin and 5% fetal bovine serum (Cytiva, Marlborough, MA). Immunodeficient NU/NU mice were used in the amyloidoma experiments described hereafter. All animal studies described herein were carried out in accordance with protocols approved by the University of Tennessee Institutional Animal Care and Use Committee and in accordance with the guidelines provided by the Office of Laboratory Animal Welfare (OLAW) and the Guide for the Care and Use of Laboratory Animals. The University of Tennessee Graduate School of Medicine is an Association for Assessment and Accreditation of Laboratory Animal Care International (AAALAC)-accredited institution.

### 2.2 Fibrils and amyloid extract

Amyloid-like fibrils composed of rVλ6Wil were prepared in PBS with 0.05% sodium azide as previously described (24). Briefly, λ6 WIL light chain variable domain monomers were purified from *e. coli* periplasmic fractions. Following lyophilization, protein was resuspended in PBS and shaken at 37°C to induce fibril formation. Fibril content was confirmed by Thioflavin T (ThT) fluorescence analysis and classical fibrillar conformation was confirmed by transmission electron microscopy. Human amyloid extracts were prepared from autopsy-derived tissues from patients with ALλ (designated CLA) or ALκ (TAL) associated amyloidosis using the water flotation method, as previously described (25). The use of human-subject–derived materials was approved by the University of Tennessee Graduate School of Medicine Institutional Review Board.

### 2.3 Isolation of primary human and murine neutrophils

Human blood was drawn from healthy, consented donors under an IRB-approved protocol (University of Tennessee Graduate School of Medicine IRB no.: 4011). Neutrophils were purified from 20 mL of peripheral blood as previously described (26). Briefly, blood was diluted 2:1 in PBS and 10 mL lymphocyte separation media (Lonza Biosciences, Walkersville, MD) was underlaid. Centrifugation was used to separate the blood into peripheral blood mononuclear cells and a neutrophil and red blood cell pellet. Red blood cells were lysed using a hypotonic lysis, yielding isolated neutrophils. Neutrophil purity (based on CD66b-FITC expression) was confirmed by flow cytometry (Northern Lights Cytometer, Cytek, Fremont, CA), and was greater than 95%. Murine neutrophils were obtained *via* intraperitoneal injection of 3% Thioglycolate (27, 28). Whole peritoneal exudate was stained with CD11b and Ly6G to identify neutrophils which phagocytosed amyloid.

### 2.4 Optical imaging of fluorophore-labeled patient extracts fibrils

A suspension of patient-derived amyloid extract (2 mg extract in 0.2 mL), containing 10% (w/w) DyLight 800-labeled (DL800) extract, was injected subcutaneously, on the dorsal flank of male and female NU/NU mice (n = 8-12). Mice, under isoflurane anesthesia, were imaged serially, over a 15-day period after injection using an iBox Scientia small animal optical imaging system (Analytik Jena, Upland, CA), using an 800-nm bandpass filter set for DL800 (2-second exposures with 1 × 1 binning). Animals were euthanized by isoflurane overdose at day 18 post injection of amyloid material, and the residual masses were harvested and fixed in 10% buffered formalin (24 h) and paraffin embedded for histologic examination.

### 2.5 Image analysis

The mean raw density (MRD) of the amyloid-associated fluorescence in the DL800 images was performed by region of interest (ROI) analysis using VisionWorks v. 8.20 (Analytik Jena, Upland, CA). Freeform ROIs encompassing the amyloid mass were manually drawn on the images by a single reviewer who was blinded to the study design. A single ROI was used for each timepoint for each individual animal in the cohort. A second ROI covering a representative amyloid-free region of the mouse was drawn and served as the mouse-specific background fluorescence (associated with autofluorescence). The final fluorescence signal intensity was calculated by subtraction of the background mean raw density from the amyloid-associated fluorescence MRD.

### 2.6 Amyloidoma area calculation

Amyloidomas were harvested 18 days post implantation. Amyloidoma area was calculated as previously described (29) using NIH ImageJ software (http://rsbweb.nih.govij). Briefly, amyloidomas were imaged with a ruler in frame for scale. ImageJ software was used to convert 1 mm into pixel number, and subsequently, area of each amyloidoma was calculated.

### 2.7 Histologic and immunohistochemical tissue staining

Sections (6 μm thick) of residual amyloid embedded in paraffin were prepared on Plus slides and stained with hematoxylin and eosin (Fisher Scientific, Waltham, MA) or alkaline Congo red solution (0.8% w/v Congo red, 0.2% w/v KOH, and 80% ethanol) for 1 h at room temperature, followed by counterstain with Mayer’s hematoxylin for 2 minutes. In order to specifically identify intact mouse neutrophils in amyloidoma sections, we performed Ly6G clone 1A8 (Fisher Scientific, Waltham, MA) staining. Neutrophil extracellular traps were histologically identified by the co deposition of the DNA-reactive dye hematoxylin [blue], the presence of transmembrane neutrophil marker LY6G, and the presence of citrullinated histone 3, in the absence of intact whole neutrophils (30).

Photomicrographs were acquired using a BZ-X700E microscope (Keyence, Atlanta, GA). Congo red fluorescence images utilized a Texas red filter. Bright-field images and were typically acquired using a 40× objective and an exposure time of 1/40 seconds, unless otherwise noted.

### 2.8 Mass spectrometry proteomic analysis of patient derived amyloid material

Analysis was performed at the clinical mass spectrometry facility at the Mayo Clinic (Rochester). Briefly, nano-flow liquid chromatography electrospray tandem mass spectrometry was performed as previously described (31, 32). Peptide spectra present in the raw data files were matched against a composite protein sequence database using three different search engines (Sequest, X!Tandem, and Mascot).

### 2.9 Transmission electron microscopy

Patient-derived amyloid material (20 μg) was placed onto a carbon coated microgrid and stained with UranyLess (Electron Microscopy Sciences, Hatfield, PA) solution for 1 minute. Samples were allowed to dry overnight. All the Transmission Electron Microscopy images were captured using the JEOL JEM 1400-FLAH TEM (JEOL USA, Inc. Peabody, Massachusetts, USA) at 120 kV with a Gatan OneView camera (Pleasanton, California, USA). Images were evaluated using ImageJ software.

### 2.10 Neutrophil depletion

***In vivo*** depletion of neutrophils was performed using anti-Ly6G (clone 1A8) (Bio X Cell, Lebanon, NH) as previously described (28). Briefly, depleting antibodies were administered (0.25 mg) every day starting at 2 days prior to amyloid implantation and then every other day for 7 days. Flow cytometry and immunohistochemistry were used to confirm neutrophil depletion.

### 2.11 Amyloidoma immunophenotyping

Human ALλ(CLA) or ALκ(TAL) extracts (2mg) were implanted subcutaneously into NU/NU mice. Amyloidomas were harvested 1 day and 8 days post implantation. Tissue was mechanically dissected and treated with type-IV collagenase for 30 minutes with shaking at 37°C. Digested samples were passed through a 70 μm filter to obtain single cell suspensions. Cells were then stained with the following antibodies: Live/Dead Aqua (Invitrogen, Thermo Fisher Scientific, Waltham, MA), CD45 (clone 30-F11), CD11b (clone M1/70), Ly6G (clone 1A8), Ly6C (clone HK1.4), F4/80 (clone BM8). All antibodies were obtained from BioLegend (San Diego, CA) unless otherwise specified. Following staining, samples were analyzed using a Cytek Northern Lights flow cytometer equipped with 405 nm and 488 nm lasers (Cytek, Fremont, CA). A Boolean gating strategy was used to remove signal from debris and dead cells, and to identify cellular populations. Data were analyzed using FlowJo software version 10.3.

### 2.12 Flow cytometric assays

For phagocytosis assays, between 1x10^5^ and 5x10^5^ primary human neutrophils or whole murine peritoneal exudate were incubated with 20 μg of patient-derived amyloid extract labeled with the pH-sensitive fluorophore pHrodo Red (20% w/w pHrodoTM red) for 1 h, 3 h, or 6 h at 37°C in a 5 mL (12mmx75mm) polystyrene tube, then analyzed for amyloid uptake using a 2-laser Northern Lights flow cytometer (Cytek, Fremont, CA). Peritoneal exudate was stained as above to identify neutrophils which phagocytosed amyloid. A Boolean gating strategy was used to identify live, single cells. Data were analyzed using FlowJo software version 10.3.

To parameterize neutrophil oxidative burst following exposure to amyloid extract, purified human neutrophils were assessed using a total ROS (reactive oxygen species) staining kit (Invitrogen, Thermo Fisher, Waltham, MA). ROS production was quantified using a Northern Lights flow cytometer (Cytek, Fremont, CA).

To analyze cytokine expression in the excised amyloidomas following neutrophil-depletion, extracted amyloidoma tissue was mechanically homogenized in 100 μL PBS and supernatants processed using the BioLegend, LEGENDplex™ mouse anti-virus response panel. Data were collected using a Northern Lights flow cytometer (Cytek, Fremont, CA). Data was analyzed using the LEGENDplex™ Data Analysis Software Suite from Qognit.

### 2.13 H_2_O_2_ treatment of amyloid

An aliquot of 100 μg of patient-derived amyloid extract or purified rVλ6WIL fibrils were incubated overnight in a 30% H_2_O_2_ 70% PBS solution (non-chelex treated), or PBS alone. Following incubation, amyloid was washed twice *via* centrifugation at 1000 x g and resuspended in PBS. Fibril content post-H2O2 treatment (or PBS control) was analyzed by Thioflavin T fluorescence emission.

### 2.14 Statistical analyses

For direct comparison of two normally-distributed variables, unpaired, two-tailed Student’s t-tests (α = 0.05) were used. Comparison of mean raw density fluorescence data between initial, peak, and subsequent time points, when normally distributed, was performed by using a mixed effect ANOVA with Tukey’s multiple comparison (α = 0.05). All statistical analyses were performed using Prism software version 9.01 (GraphPad Software Inc., San Diego, CA).

### 2.15 Ethics statement

All patient-derived tissue samples were used in accordance with an Institutional Review Board-approved application. Animal studies were approved by the University of Tennessee Institutional Animal Care and Use Committee and were performed in accordance with the guidelines provided by OLAW and the Guide for the Care and Use of Laboratory Animals. The University of Tennessee Medical Center animal program is AAALAC-i-accredited.

## 3 Results

### 3.1 Comparison of ALλ(CLA) and ALκ(TAL) amyloid extracts

**Figure 1:**
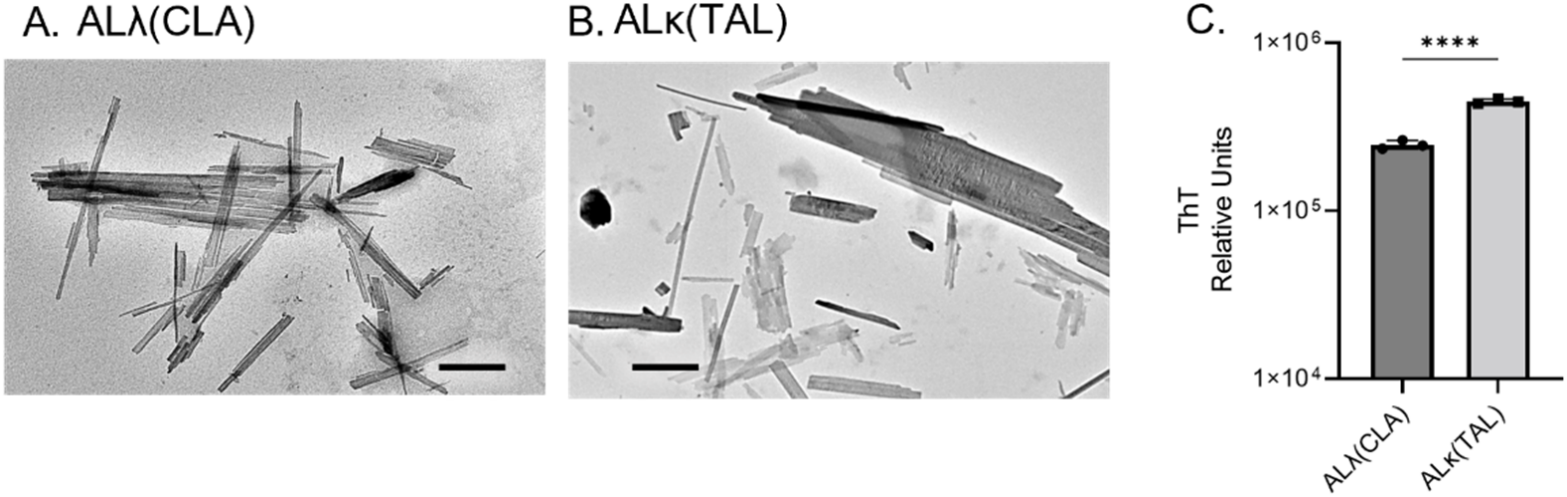
Comparison of ALλ(CLA) and ALκ(TAL) patient-derived material. Transmission electron microscopic images of indicated patient extract **(A, B)**, black scale bars represent 500nm. ThT fluorescence indicative of total fibril content **(C)**, 5μg of extract was analyzed. Statistical significance was determined by unpaired Student’s t-test ****p<0.0001.

Both human AL extracts were composed of sheets of linearly aligned, unbranching fibrils, when imaged using transmission electron microscopy. In addition, single, non-twisting, fibrillar structures were present in both samples. Both ALλ(CLA) and ALκ(TAL) appeared ultrastructurally comparable (**Figures 1A, B**). Addition of ThT resulted in similar fluorescence emission at 490 nm, characteristic of the presence of amyloid fibrils, although ALκ(TAL) exhibited significantly greater fluorescence emission suggesting the presence of either more amyloid fibrils per unit mass of extract, more ThT binding sites due to differences in fibril morphology, or higher order aggregation states **Figure 1C**.

Mass spectrometric analysis of the amyloid extracts demonstrated near-identical composition of the materials with respect to t known amyloid-associated accessory molecules (except for the presence of histone H2A in the ALκ preparation). Differences in the number of recovered peptides, potentially reflects discordance in their relative abundance in the extracts (**Table 1**).

**Table 1:** Mass spectrometry data from ALλ(CLA) or ALκ(TAL) patient tissue.

#	Identified Protein	ALλ(CLA)	ALκ(TAL)	#	Identified Protein	ALλ(CLA)	ALκ(TAL)
1	Igκ constant chain		4002	16	Pelamin-A/C	1	119
2	Igκ variable chain		444	17	Histone H2A-T1H	55	639
3	Igλ constant chain	2472		18	HSPG	537	175
4	Igλ variable chain	457		19	Fibronectin	48	342
5	ApoA-IV	873	51	20	Tubulin α-chain	6	41
6	ApoE	725	1376	21	Tubulin β-chain	4	82
7	SAP	261	408	22	β-hemoglobin	278	56
8	Serum albumin	28	89	23	Collagen-α3	414	913
9	Myosin-9	23	88	24	Histone H3	5	173
10	Histone H2B-T1K	85	853	25	Complement C3	306	111
11	Vimentin	50	840	26	Complement C1q	51	65
12	Actin	149	786	27	Histone H2A-T1C		245
13	Histon H4	60	939	28	Filamin-A	2	11
14	Vitronectin	95	1572	29	Tenascin	26	10
15	Clusterin	1265	1893	30	Collagen-α2	182	32

Amyloidogenic proteins identified are listed first, followed by the most abundant proteins. Numbers indicate the number of peptide spectra observed for each protein.

**Figure 2:**
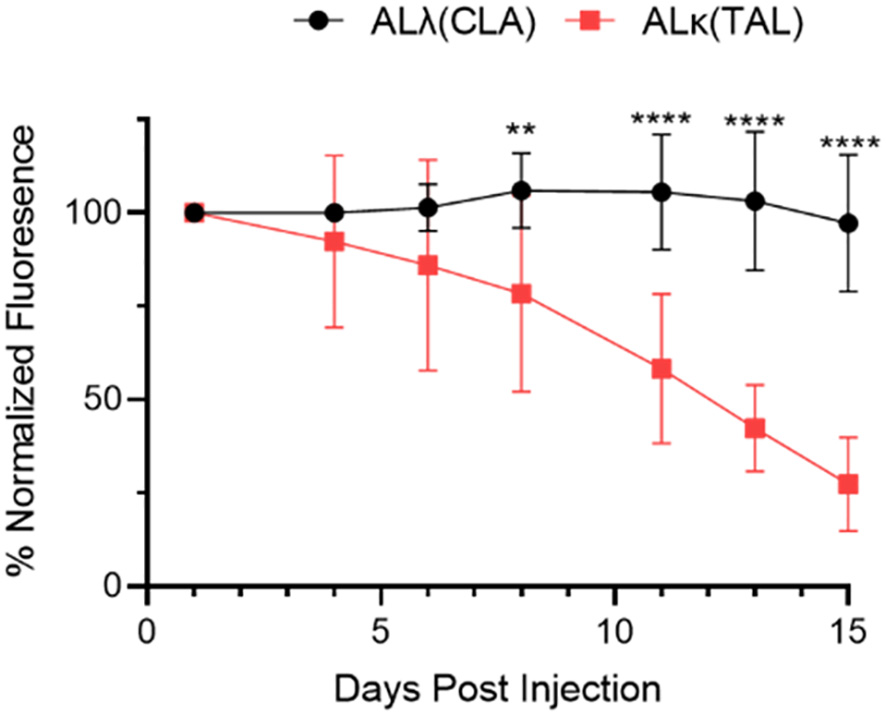
Quantification of amyloidoma-associated Dylight800 fluorescence intensity as normalized fluorescence. Data are representative of 2 experiments with n=8-12 animals per group. Mean and standard deviation at each time point shown. Data were analyzed using Student’s t-test at each time point to determine significance **, p<0.01; ****, p<0.001.

The clearance of both AL amyloid extracts implanted subcutaneously in NU/NU mice, based on the decrease in DL800 fluorescence emission, was shown to be significantly slower for ALλ(CLA) as compared to ALκ(TAL) amyloid extract (**Figure 2**). Relative to the fluorescence emission at Day 1, by Day 15 post-injection the fluorescence emission associated with the ALλ(CLA) amyloidoma had not decreased, whereas that from ALκ(TAL) was ~75% reduced.

### 3.2 Immunological evaluation of cellular infiltrates into ALλ(CLA) or ALκ(TAL) amyloidomas

**Figure 3:**
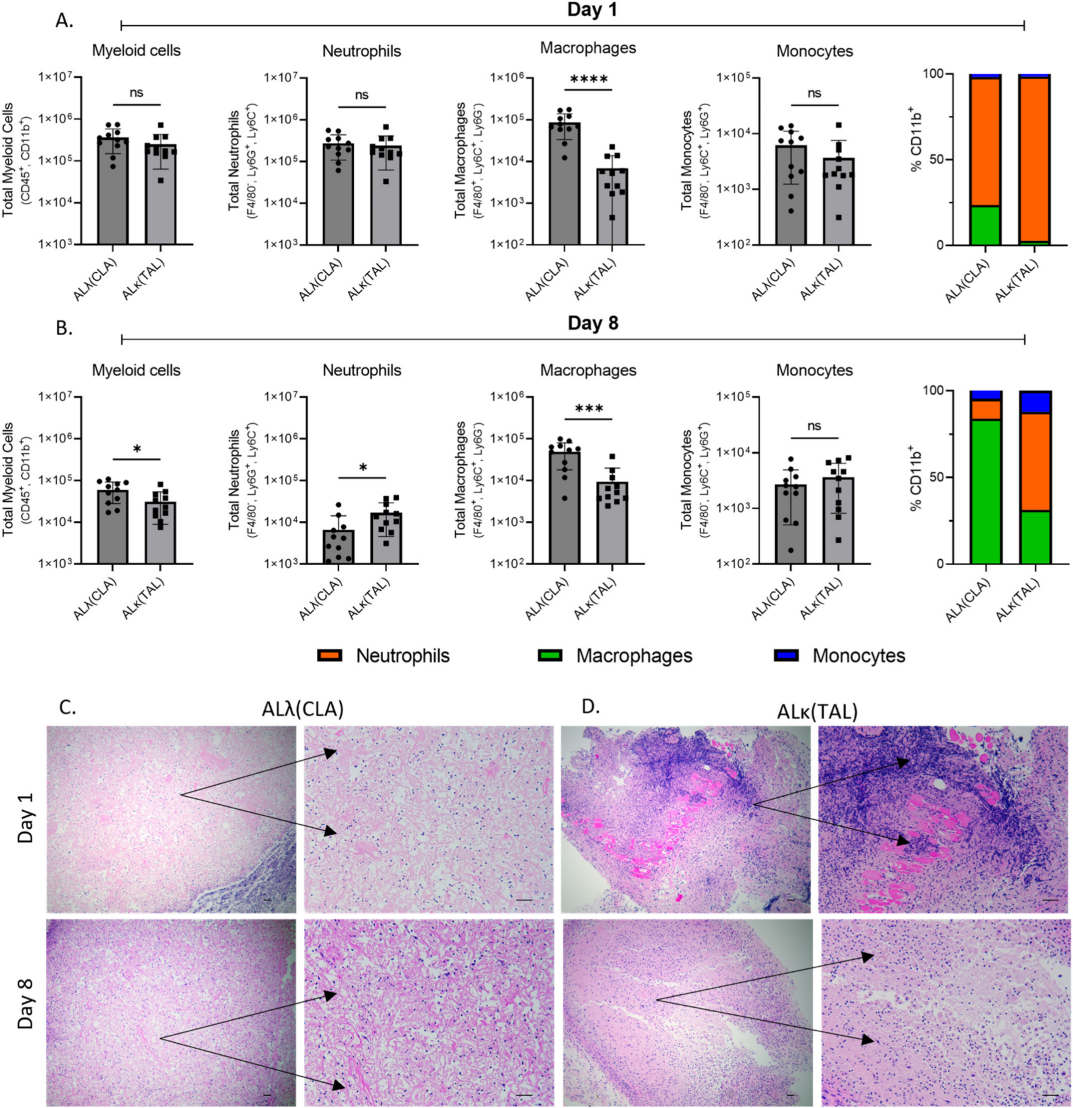
Immunophenotypic evaluation of immune infiltrate into ALλ(CLA) or ALκ(TAL) amyloidomas. Flow cytometry was used to identify single cell suspensions isolated from indicated tissues Day 1 **(A)** and Day 8 **(B)** post amyloid implantation. Cell surface markers used to identify each indicated population are detailed on the y-axis of each panel. The gating strategy used for analysis can be found in **Supplementary Figure 1**. Excised amyloidoma tissues sections were stained with H&E to identify immune cell infiltrates **(C, D)**. Images are representative of 3-4 mice per group. Black scale bars represent 50μm. Data represent the mean ± standard deviation from one experiment which is representative of two experimental replicates, with n=5-6 animals per group per experiment. Statistical significance was determined by Student’s t test, ∗p < 0.05, ∗∗∗p < 0.001, ∗∗∗∗p < 0.0001. ns, not significant.

Since ALλ(CLA) and ALκ(TAL) amyloid clearance rates differed *in vivo*, we sought to determine whether immunological changes within the two patient-derived amyloidomas could be observed. Amyloidomas were harvested from NU/NU mice 1 day and 8 days post implantation and immunophenotyping was performed to identify immune cell infiltrates (See **Supplementary Figure 1** for the gating strategy used for analysis). Phenotypically, 1 day post amyloid implantation there was no difference in the number of live myeloid cells (CD45^+,^CD11b^+^) isolated from either the ALλ(CLA) and ALκ(TAL) amyloidomas. However, the composition of the myeloid cell compartment varied as the total number of macrophages (CD45^+,^CD11b^+^, Ly6C^+^, Ly6G^-^, F4/80^+^) identified in ALλ(CLA) amyloid was significantly greater than that in seen in ALκ(TAL) amyloid. There was no difference in the number of live neutrophils (CD45^+,^CD11b^+^, Ly6C^+^, Ly6G^+^, F4/80^-^) or monocytes (CD45^+,^CD11b^+^, Ly6C^+^, Ly6G^-^, F4/80^-^) identified between ALκ and ALλ amyloidomas at 1 day post amyloid implantation. Eight days post amyloid implantation, ALλ(CLA) amyloidomas contained significantly more live myeloid cells as well as a continued significant increase in macrophages compared to ALκ(TAL). However, ALκ(TAL) exhibited significantly more neutrophils compared to ALλ(CLA) (**Figure 3B**). There was no difference in monocyte content 8 days post amyloid implantation. Finally, overall myeloid cell composition between ALλ(CLA) and ALκ(TAL) amyloidomas was drastically different (**Figures 3A, B**, far right panels %CD11b).

**Figure 4:**
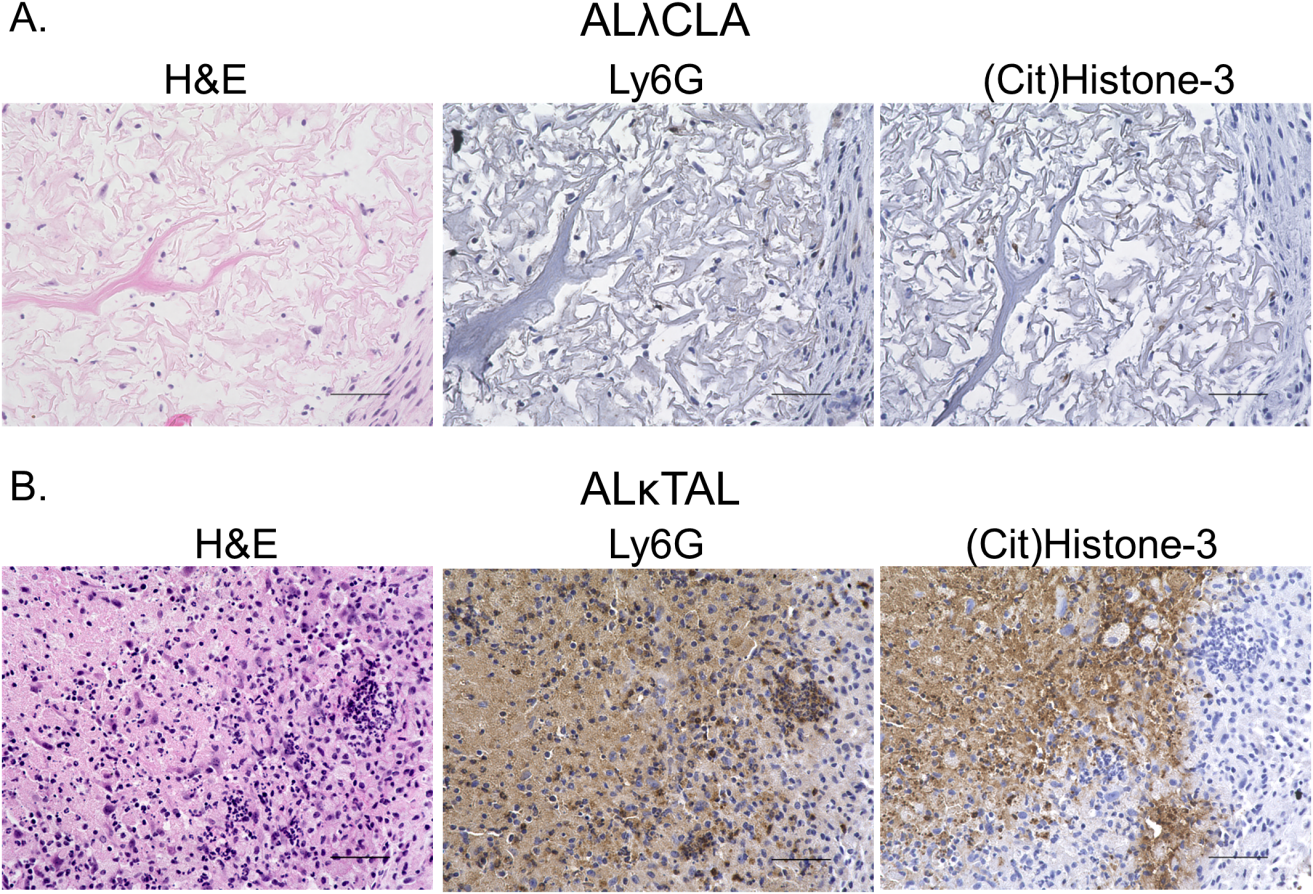
Spatial analysis of intra-amyloid neutrophil recruitment. Immunohistochemistry was used to specifically analyze neutrophils in tissues 8 days post the implantation of either ALλ(CLA) **(A)** or ALκ(TAL) **(B)** amyloidomas. H&E staining was performed to identify polymorphonuclear cells and other immune infiltrate. Ly6G staining was used to identify intact neutrophils and neutrophil membrane remnants. Citrullinated histone 3 staining was used to identify neutrophil extracellular traps. Data is representative of two experimental replicates with 5 animals per group. Scale bars = 50μm.

To spatially interrogate intra-amyloid immune cell distribution, tissue sections were analyzed 1 day and 8 days post ALλ(CLA) and ALκ(TAL) amyloidoma implantation. Staining of the amyloidomas excised at day 1 post injection with H&E showed pronounced infiltration of polymorphonuclear cells (*i.e.*, neutrophils) throughout the ALκ(TAL) amyloidoma with profound DNA deposition (**Figure 3D** black arrows). There was no observable DNA deposition observed in ALλ(CLA) amyloidomas and the presence of neutrophils was markedly reduced relative to the ALκ(TAL) amyloidoma (**Figure 3C** black arrows. Subsequent evaluation of amyloidomas harvested 8 days post implantation indicated that ALλ(CLA) amyloid did have immune infiltrate; however, ALκ material again contained identifiable polymorphonuclear cells, large macrophage-like cells, and multinucleated giant cells. Next, we subjected ALλ(CLA) and ALκ(TAL) amyloidomas harvested 8 days post implantation to a combinatorial staining regime to specially analyze neutrophils by combining H&E staining, the neutrophil marker, Ly6G, and the neutrophil extracellular trap marker, citrullinated histone 3. ALλ(CLA) amyloidomas were devoid of neutrophils 8 days post amyloid implantation as evidence by both H&E staining and Ly6G staining (**Figure 4A**, left and middle panels). In contrast, ALκ(TAL) amyloidomas contained a plethora of Ly6G staining including both intact neutrophils and diffuse intra-amyloidoma Ly6G staining, suggesting neutrophil death (**Figure 4B**). The diffuse intra-amyloidoma Ly6G staining co-localized with pronounced citrullinated histone 3 indicating the presence of neutrophil extracellular traps. Importantly, co-citrullinated histone 3 and Ly6G staining was not observed in the intact neutrophil population, (**Figure 4B** right panel).

Based on the amount of observable NETosis and abundance of identifiable cellular/nuclear debris in the ALκ amyloidomas, we reexamined the flow cytometry data in **Figures 3A, B** this time including non-viable cells which were identified using an amine reactive viability dye (traditionally removed using a standard Boolean gating strategy **Supplementary Figure 1**). The proportion of viable CD45^+^ immune cells compared to the total population of immune cells was 2.5-fold (p ≤ 0.0001) lower in ALκ(TAL) 1 day following amyloid implantation and at 8 days post implantation of the amyloid ALκ(TAL) amyloidomas contained 3.75-fold (p ≤ 0.0001) fewer viable immune cells compared to ALλ(CLA) (**Supplementary Figure 2**). Inclusion of non-viable cells in our analysis identified significantly more neutrophils at both day 1 (*p*=0.0044, 2.25-fold) and day 8 (*p*=0.0022, 9.75-fold) post amyloid implantation in ALκ(TAL) compared to ALλ(CLA) (**Supplementary Figure 2**). Examination of the remaining immune cells was not significant when including non-viable cells, but the number of identified monocytes in ALκ(TAL) amyloidomas trended towards an increase and the number macrophages isolated from either amyloidoma was virtually identical (**Supplementary Figure 2**).

**Figure 5:**
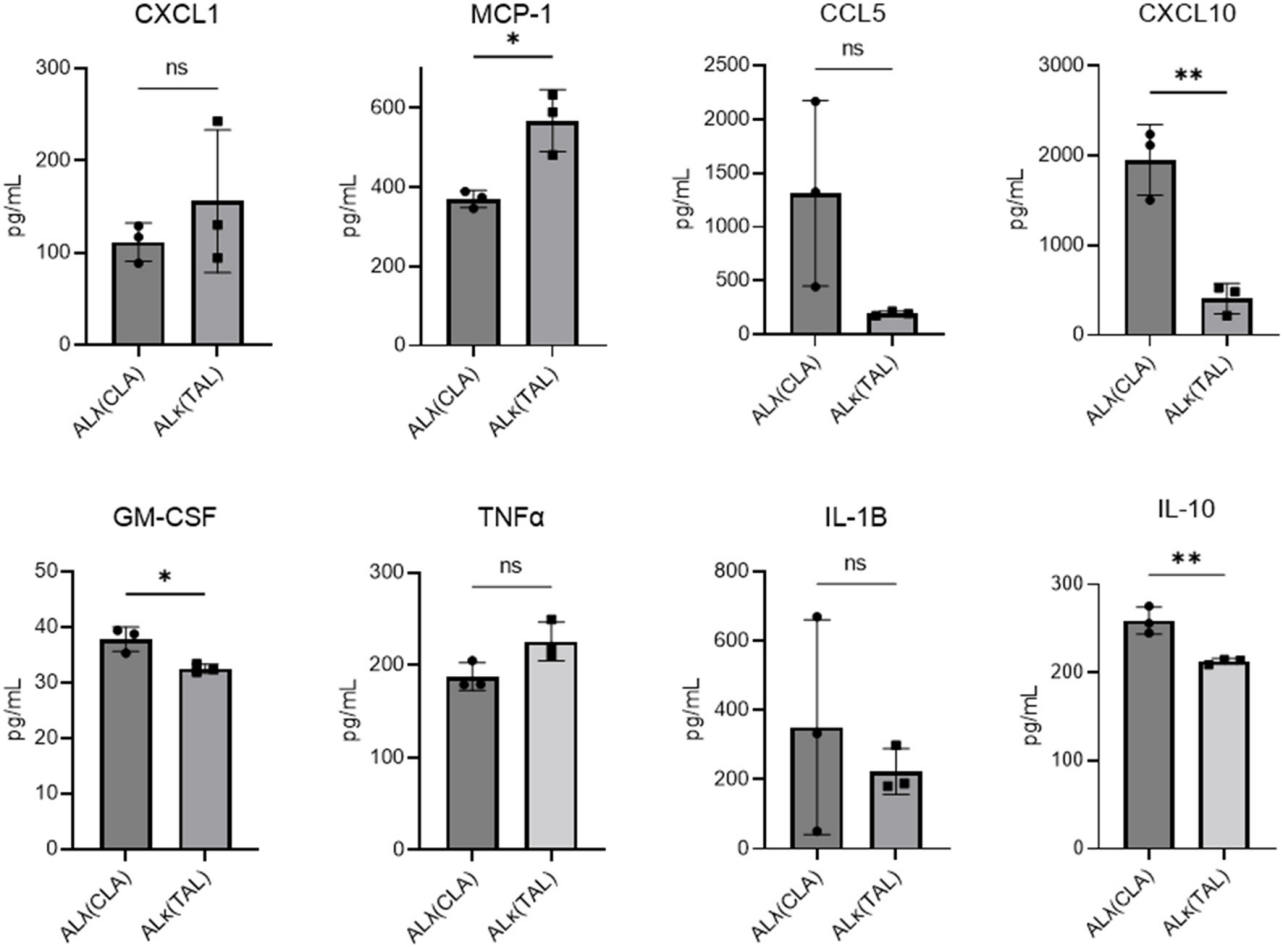
Amyloidoma cytokine expression in ALλ(CLA) or ALκ(TAL) amyloidomas. NU/NU mice were treated as in **Figure 3** and implanted with ALκ(TAL) or ALλ(CLA) amyloidomas. 8 days following amyloid implantation amyloidomas were harvested and cytokine expression determined using a BioLegend, LEGENDplex™ panel. n=1 with 3 mice per group. Statistical significance was determined by unpaired Student’s t-test *p<0.05, **p<0.01. ns, not significant.

With the stark differences in immune cell infiltrates between ALλ(CLA) and ALκ(TAL) amyloidomas we sought to evaluate their immunological activation status. Using the BioLegend, LEGENDplex™ cytokine expression platform we analyzed cytokine expression at 8 days post amyloid implantation. ALλ(CLA) amyloidomas contained significantly more CXCL10, GM-CSF (granulocyte monocyte-colony stimulating factor), and IL-10, with more expression of CCL5 trending towards significance (*p*=0.0882) (**Figure 5**). ALκ(TAL) amyloidomas had significantly more expression of monocyte chemoattractant protein-1 (MCP1; CCL2), and more expression of tumor necrosis factor-alpha trending towards significance (*p*=0.0627) (**Figure 5**).

### 3.3 Evaluation of the neutrophil-amyloid relationship

**Figure 6:**
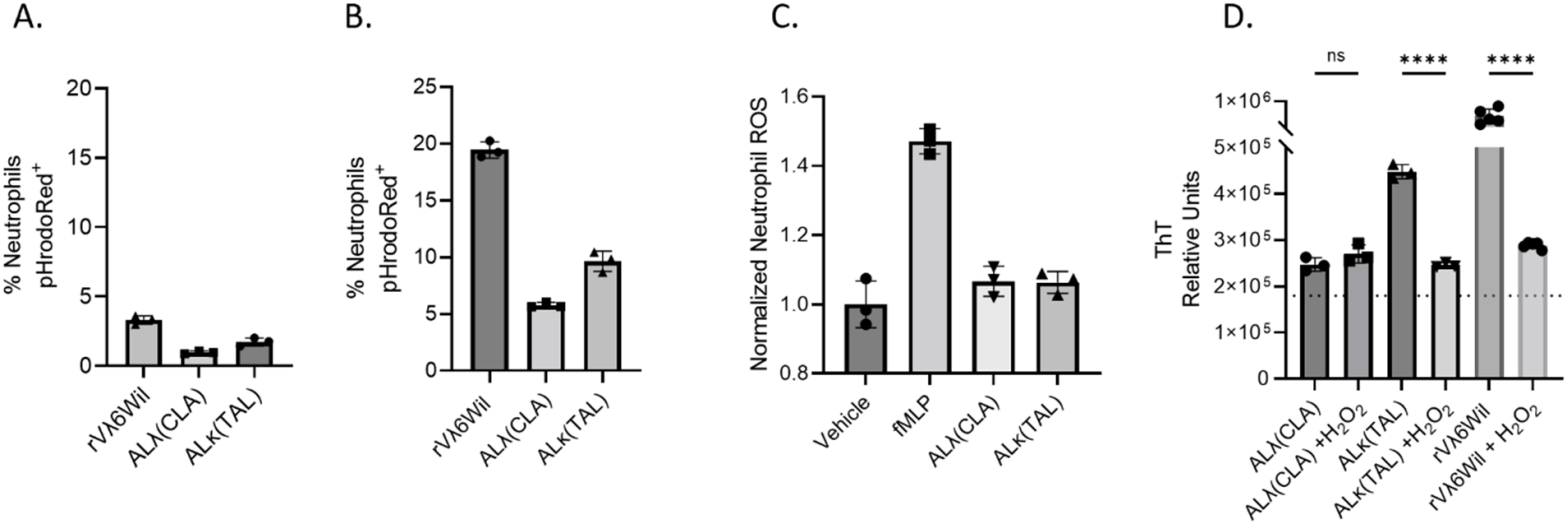
Evaluation of the neutrophil-amyloid relationship. Flow cytometry analysis of human neutrophil **(A)** or thioglycolate-elicited murine neutrophil **(B)** phagocytosis of pHrodoRed-labeled ALλ(CLA) extract or ALκ(TAL) extract. Primary neutrophil ROS productions was analyzed by flow cytometry in the presence of indicated amyloid material. **(C)**. Effect of H_2_O_2_ treatment of amyloid extract and fibril degradation **(D)**. Statistical significance was determined by unpaired Student’s t-test ****p<0.0001.

We analyzed neutrophil activity *ex vivo* following exposure to amyloid extract and amyloid-like synthetic fibrils. To assess the ability of neutrophils to directly phagocytose ALλ(CLA) extract, ALκ(TAL) extract, or purified synthetic rVλ6WIL fibrils, pHrodoRed-labeled substrates were exposed to either human or mouse neutrophils *in vitro* and analyzed by flow cytometry. In this *ex vivo* system, purified human neutrophils would not or could not phagocytose any amyloid material (**Figure 6A**). Also murine neutrophils from whole thioglycolate-elicited peritoneal exudate did not phagocytose amyloid extracts and amyloid-like fibril phagocytosis was minimal (**Figure 6B**). We next evaluated the reactive oxygen burst of human neutrophils following exposure to the amyloid substrates and, as a positive control, to formylated-met-leu-phe peptide (fMLF). Neither amyloid extract induced an oxidative burst *in vitro* (**Figure 6C**). However, since *in vivo* amyloidoma conditions are more complex than *in vitro* conditions, we questioned whether, if generated *in vivo*, what effect reactive oxygen species (*e.g.*, H_2_O_2_) would have on the amyloid extracts and synthetic fibrils. Hydrogen peroxide treatment of both rVλ6Wil fibrils and ALκ(TAL) extract significantly reduced the ThT fluorescence emission associated with the substrates. In contrast, ALλ(CLA) amyloid was refractory to H_2_O_2_ treatment, and no significant change in ThT fluorescence emission was observed (**Figure 6D**).

### 3.4 Neutrophil depletion delays amyloid resolution *in vivo*

**Figure 7:**
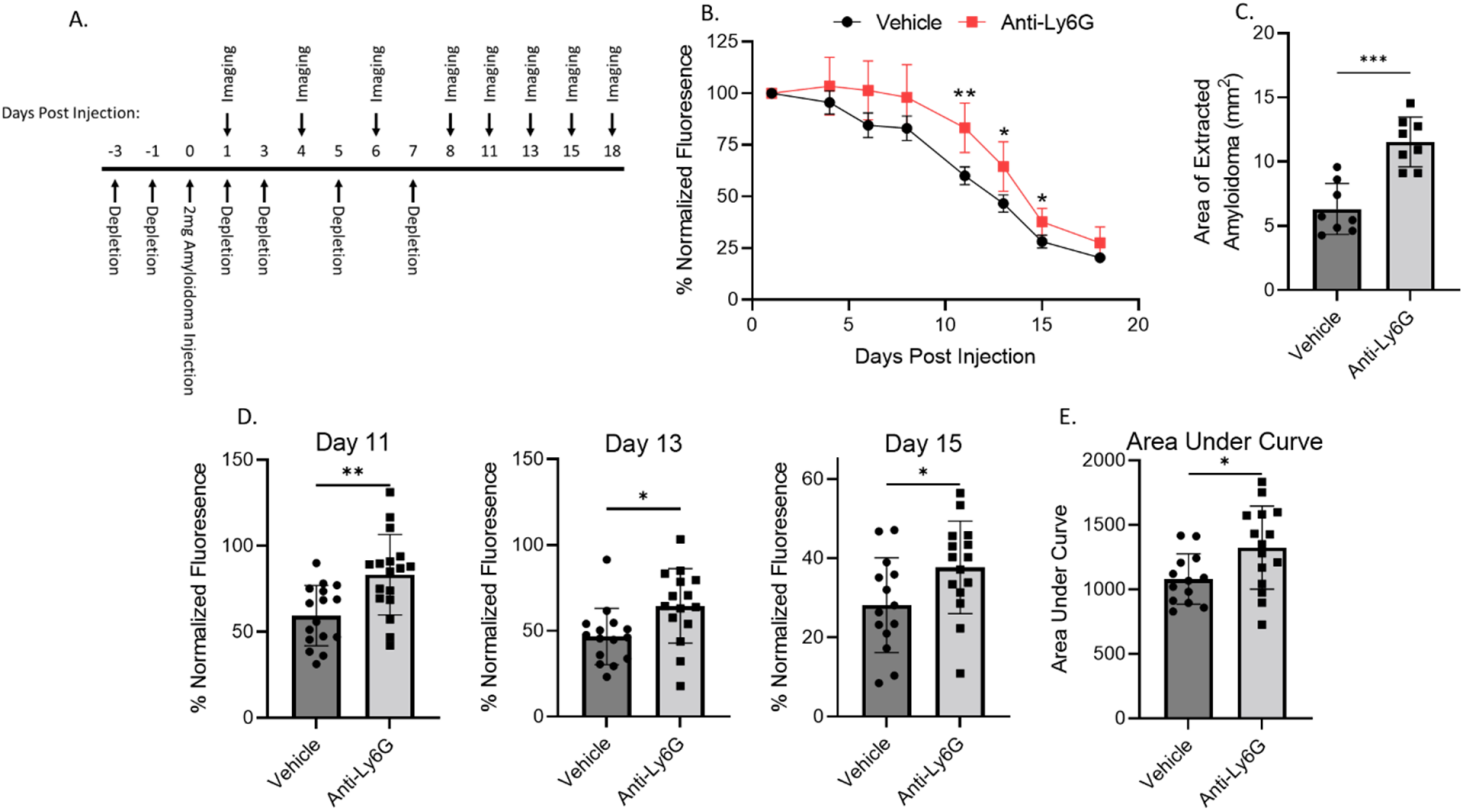
Neutrophil depletion impedes ALκ(TAL) resolution *in vivo*. Immunodeficient mice were implanted with Dylight800 labeled ALκ(TAL) amyloidomas and fluorescence measured over time **(A, B, D, E)**,. 18 days post-implantation, amyloidomas were extracted, and amyloid area was determined by ImageJ **(C)**. Mice received either the anti-Ly6G (1A8) neutrophil depletion antibody or vehicle control treatment. Data is representative of 2 experiments with 8-12 mice per group. Statistical significance was determined by unpaired Student’s t-test *p<0.05, **p<0.01, ***p<0.001.

Given the neutrophil influx and resultant NETosis in the ALκ(TAL) amyloidoma and their potential impact on the amyloid mass, we assessed the impact of neutrophil depletion on ALκ(TAL) dissolution *in vivo* (**Figure 7**). Neutrophil depletion, accomplished using the anti-Ly6G (1A8) antibody and confirmed by flow cytometry and histological analysis (**Supplementary Figure 3**), significantly delayed ALκ(TAL) amyloidoma clearance in NU/NU mice as evidenced by changes in DL800 fluorescence intensity over 18 days post amyloid injection (**Figures 7A, B**). The amyloid mass at days 11, 13, and 15 post-injection was significantly less (as measured *via* fluorescence) in untreated mice as compared to neutrophil-depleted animals (**Figures 7B, D**). Furthermore, measurement of the area of the residual amyloid masses, harvested from the mice at necropsy, showed ~2-fold larger amyloid lesions in mice treated with neutrophil-depleting antibodies (**Figure 7C**).

**Figure 8:**
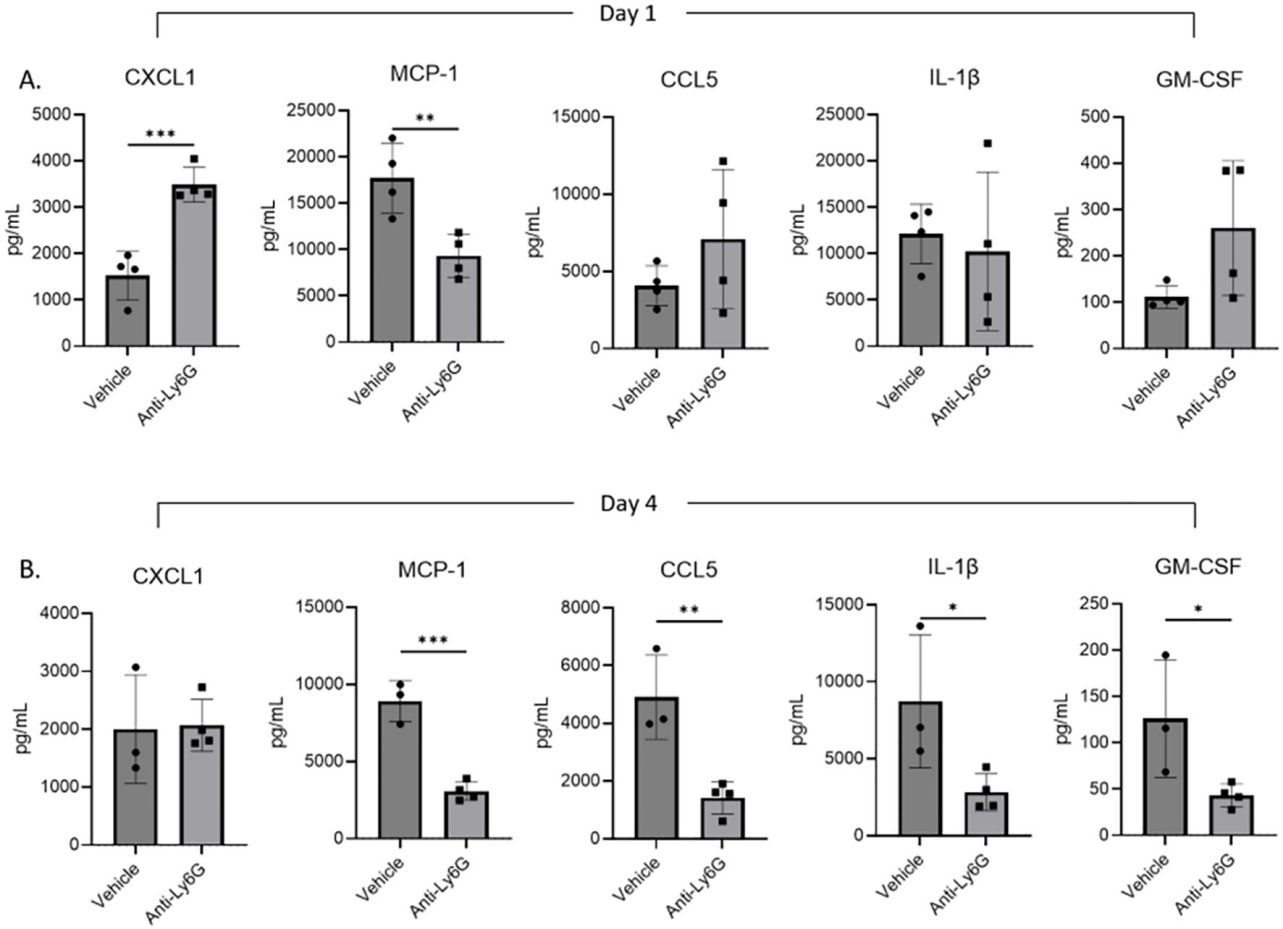
Amyloidoma cytokine expression in neutrophil depleted or non-depleted mice. NU/NU mice were treated as in **Figure 5A** and implanted with ALκ(TAL) amyloidomas. 1 **(A)** and 4 **(B)** days following amyloid implantation amyloidomas were harvested and cytokine expression determined using a BioLegend, LEGENDplex™ panel. n=1 with 3-4 mice per group. Statistical significance was determined by unpaired Student’s t-test *p<0.05, **p<0.01, ***p<0.001.

To further interrogate the mechanisms by which neutrophils may impact ALκ(TAL) clearance, we monitored the immune activation status of the ALκ(TAL) amyloid excised from neutrophil-depleted and naive mice. Cytokine and chemokine expression was measured 1- and 4-days post implantation by multiparameter flow cytometric ELISA-based assays. Day 1 and day 4 were chosen as both were within the active neutrophil depletion window (**Figure 8A**) before depletion pressure was removed. Amyloid from neutrophil-depleted mice had significantly more CXCL1, a neutrophil chemokine, compared to amyloid from non-depleted animals, which had significantly more expression of MCP-1. No significant differences were observed in amyloid associated CCL5 (RANTES), IL-1β, or GM-CSF. At day 4, naïve animals had significantly more expression of MCP-1, CCL5, as well as the inflammatory mediators IL-1β and GM-CSF (**Figure 8B**) compared to amyloid excised from neutrophil-depleted mice.

**Figure 9:**
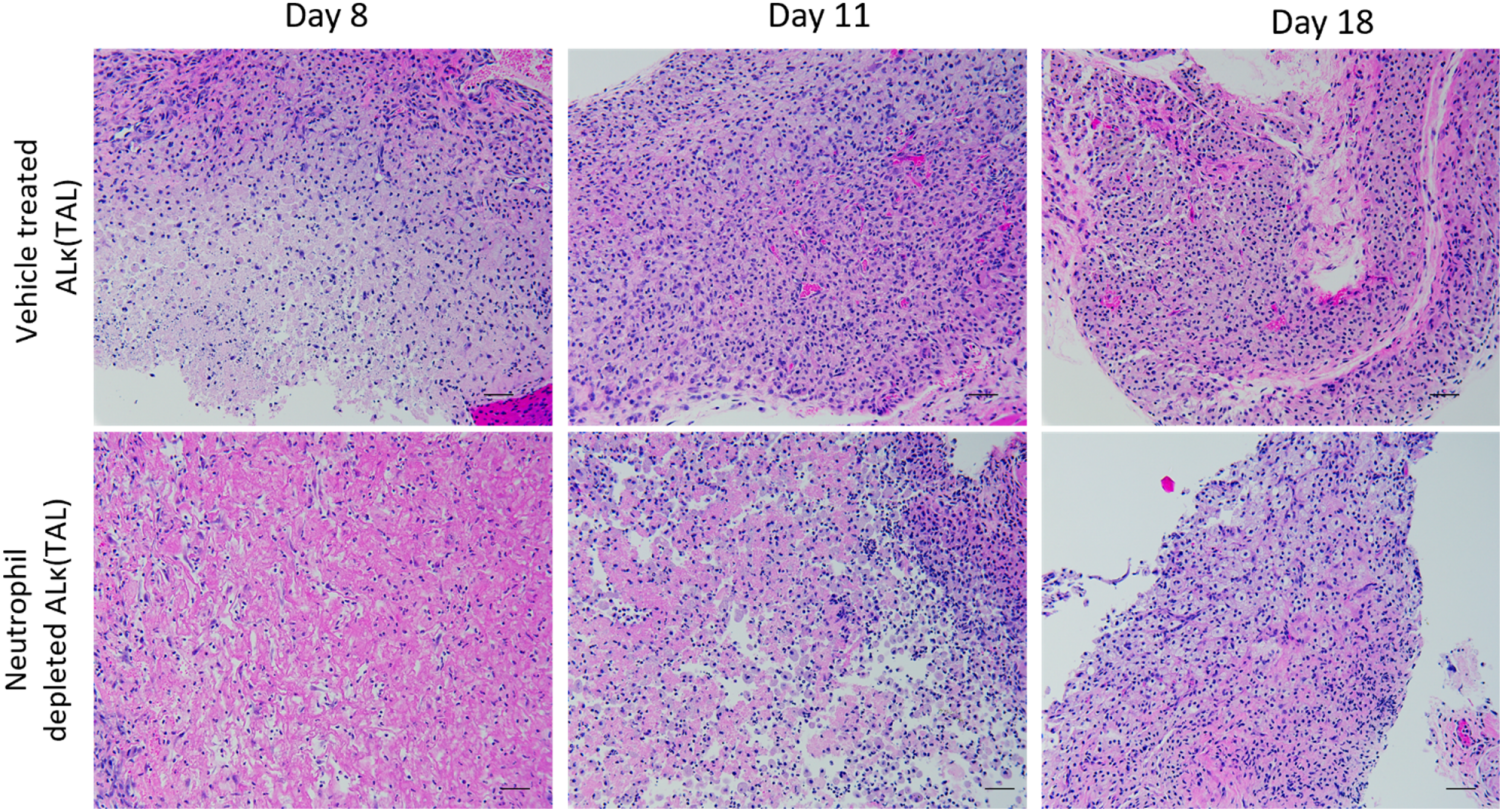
Neutrophil depletion delays amyloidoma microenvironment maturation. Excised amyloidoma tissues sections were stained with H&E to identify immune cell infiltrates. Images representative of n=4 mice per group. Black scale bars represent 50μm.

Histological evaluation of ALκ(TAL) amyloid resected from neutrophil-depleted NU/NU mice or non-treated NU/NU mice on days 8, 11, and 18 following depletions indicate a delay in neutrophil infiltration of the amyloidoma in mice depleted of neutrophils compared to untreated-mice. Non-depleted mice had a concomitant accumulation of multinucleated giant cells as well as polymorphonuclear cells within the amyloid deposits at earlier time points (Day 8) compared to neutrophil-depleted mice (Day 11) (**Figure 9**). Furthermore, the immunological landscape and amyloidoma structure of neutrophil-depleted day 8 ALκ(TAL) amyloidomas and day 8 ALλ(CLA) amyloidomas (**Figures 3C, D**) were visually similar.

## 4 Discussion

Systemic amyloidosis is characterized by the aggregation of amyloidogenic precursor proteins into highly ordered fibrils that deposit in organs and tissues, resulting in progressive organ dysfunction, severe morbidity, and death (1, 2, 4). Current standards of care for AL amyloidosis, the second most prevalent form of systemic amyloidosis, seek to abolish precursor protein production (33). While vital, these therapies do not directly address the removal of preexisting amyloid deposits. As tissue amyloid burden correlates with organ dysfunction and disease morbidity (11–14), curative therapies will likely require combinatorial approaches addressing both inhibition of amyloid precursor protein production and tissue amyloid removal (34). Several anti-amyloid therapeutics, amyloid-reactive antibodies, are in development to stimulate immune-mediated amyloid removal, but these reagents are not yet approved for clinical use (35–39).

Current models of immune-mediated amyloid clearance suggest that macrophages are vital to amyloid dissolution (40–42). Recently, we assayed the propensity of macrophages to phagocytose 15 patient-derived amyloid extracts (five ALκ extracts, five ALλ extracts, and five ATTR extracts) following collagen degradation, which significantly enhanced uptake in 14 of the 15 extracts evaluated (22). Interestingly, *in vivo* experiments indicated that degradation of amyloid-associated collagen expedited clearance of amyloid which coincided with an influx of both macrophages and neutrophils into the subcutaneous amyloid mass. These data, together with observations which indicated that neutrophils influence amyloid resolution following antibody therapy in a murine model of amyloidosis (23), suggest that neutrophils may participate in amyloid removal in the localized amyloidoma murine model. However, the mechanisms by which these cells contribute to amyloid clearance has not been examined. Here using a murine model of human AL amyloidomas, we assessed the role of neutrophils in amyloid resolution independent of anti-amyloid antibody intervention.

We have identified certain human AL amyloid extracts, (*e.g.*, ALκ(TAL)), which are rapidly cleared when injected subcutaneously into immunocompromised mice. In contrast, the ALλ(CLA) extract represents an extract that is resistant to clearance in the mouse. These two patient-derived extracts are structurally and compositionally similar based on microscopic, and mass spectrometric analysis. It is important to note that these two extracts are representative of either a quickly removed amyloid extract or an amyloid extract which is refractory to clearance. AL subtype is not thought to influence amyloid clearance as amyloids from both subtypes have been demonstrated to be efficiently taken up by macrophages (22) and cleared in this mouse model (23). Interestingly, following implantation into NU/NU mice, the immunological environments which develop in each of the two amyloidomas are significantly different. A rapid, extensive, and sustained neutrophil influx is seen with ALκ (TAL) amyloid, followed by the eventual recruitment of macrophages (**Figure 3**). In contrast, ALλ(CLA) injection results in recruitment of macrophages at early time points post injection, with neutrophils only observed on day 1 post-implantation. Why the ALκ and the ALλ extracts induce differential immune environments is unknown. However, recent observations suggest that collagen could play a role (22). Furthermore, the substantial presence of non-viable immune cells within ALκ (TAL) amyloidomas compared to ALλ(CLA) could be representative of amyloid induced cellular toxicity as we recently reported that there is a direct inverse correlation between cell viability and amyloid uptake (52).

These observations and the innate neutrophil influx into implanted ALκ(TAL) amyloid led us to further interrogate the neutrophil-amyloid relationship. *In vitro* studies indicated that human neutrophils could not or would not phagocytose either patient-derived amyloid extracts or synthetic amyloid-like fibrils, and activated thioglycolate-elicited murine neutrophils were able to marginally engulf only synthetic amyloid-like fibrils (**Figure 5**). However, neither human nor mouse neutrophils are able to phagocytose these materials as effectively as species specific macrophages (22). Therefore, we queried whether neutrophil mediators could influence amyloid degradation as a putative first step in amyloid clearance. Treatment of both synthetic fibrils and ALκ(TAL) extract with H_2_O_2_ induced changes in amyloid structure that resulted in a decrease in ThT fluorescence, possibly indicating dissolution of fibrils or disruption of the β-sheet fibril ultrastructure. In contrast, ALλ(CLA) ThT incorporation was unaffected by this treatment. Subcutaneous ALκ(TAL) amyloidomas contained a plethora of neutrophil extracellular traps (NETs), indicating activation of the neutrophils in the amyloid; however, these were not observed in ALλ(CLA) lesions, suggesting that neutrophils become differentially activated when entering in ALκ(TAL) lesions compared to ALλ(CLA) lesions (**Figures 3**, **4**). Neutrophil extracellular traps are known to decorate the surface of bacterial pathogens to aid in pathogen clearance. These structures also contain reactive oxygen species (ROS) as well as proteolytic enzymes such as neutrophil elastase all of which function to degrade and flag extracellular pathogens for clearance (26, 43–45). The presence of NETs in amyloidomas may serve to opsonize amyloid for phagocytosis by macrophages, and other professional phagocytes. Additionally neutrophil activation does occur *in vivo* (as evidenced by NETosis), which cannot occur without the local accumulation and production of ROS (46, 47).

To evaluate the importance of neutrophils in the clearance of ALκ(TAL) amyloid, neutrophil depletion experiments were performed. Neutrophil depletion both significantly delayed ALκ(TAL) clearance compared to non-depleted animals (**Figure 6**), and the amyloid from neutrophil-depleted mice had significantly lower expression of inflammatory mediators such as MCP-1, CCL5, IL-1β, and GM-CSF (**Figure 7**). Moreover, amyloidomas from neutrophil depleted mice had enhanced expression of CXCL-1 a potent neutrophil chemokine indicating the potential need of this cell type in amyloid clearance. The reduced expression of MCP-1, CCL5, IL-1β, and GM-CSF coincided with, and may have contributed to, the delayed infiltration of macrophages into ALκ(TAL) amyloid masses (**Figure 8**). Taken together, these observations suggest that neutrophils may not directly clear amyloid through phagocytosis, but rather they function indirectly by disrupting the otherwise proteolytically-resistant amyloid, opsonize local amyloid by NETosis, and enhance macrophage recruitment and polarize their differentiation into multinucleated giant cells, which may be crucial for amyloid clearance (48, 49).

While these data suggest that neutrophils can help mediate human AL amyloid clearance in a mouse amyloidoma model, neutrophils are not typically found associated with tissue biopsies from patients with amyloidosis. This could be due to our model inducing a foreign body response which is known to cause an initial wave of neutrophil recruitment followed by macrophage influx into or around the foreign body, such as implanted amyloid (50, 51), or amyloid mediated cellular toxicity. However, we have exploited the foreign body response and identified a novel cell type that may be utilized to enhance the therapeutic removal of tissue amyloid. Currently, there are no anti-amyloid therapeutics specifically designed to induce the recruitment of neutrophils to amyloid and induce immune activation of these cells. We posit that neutrophils when appropriately recruited and activated could induce the macrophage-mediated clearance of tissue amyloid in patients. Here, we provide data that supports this hypothesis and indicates that neutrophils can contribute to effective amyloid clearance in preclinical murine amyloidoma models.

## Data Availability

The raw data supporting the conclusions of this article will be made available by the authors, without undue reservation.
