# Supplementary material for: Neutrophils enhance the clearance of systemic amyloid deposits in a murine amyloidoma model
Source: Front Immunol. 2024 Nov 12;15:1487250. doi: 10.3389/fimmu.2024.1487250 (PMC11588727; doi:10.3389/fimmu.2024.1487250)
Supplement: Supplementary file 1 [file DataSheet1.docx]

Supplementary Material

# Supplementary Figures


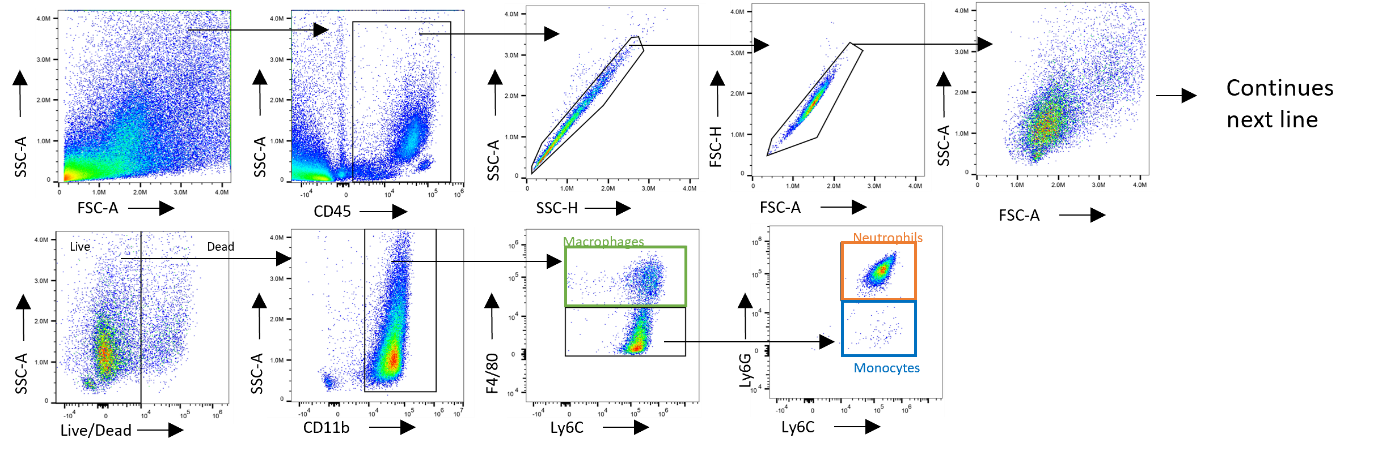


**Supplemental Figure 1 Gating strategy used to identify immune cell infiltrates into amyloid lesion.** Whole amyloidoma homogenates were used for analysis. Cell surface markers used to identify each indicated population are detailed on the y- or x-axis of each panel.

**Supplementary Figure 2 Inclusion of nonviable cells in the immunophenotypic evaluation of immune infiltrate into ALλ(CLA) or ALκ(TAL) amyloidomas.** Flow cytometry was used to identify single cell suspensions isolated from indicated tissues Day 1 (top panels) and Day 8 (bottom panels) post amyloid implantation. Cell surface markers used to identify each indicated population are detailed on the y-axis of each panel. The gating strategy used for analysis can be found in Figure S1. Presented data represent the mean ± standard deviation from one experiment which is representative of two experimental replicates, with 5 animals per group. Statistical significance was determined by Student’s t-test, ∗*p < 0.01, ∗∗∗∗p < 0.0001.


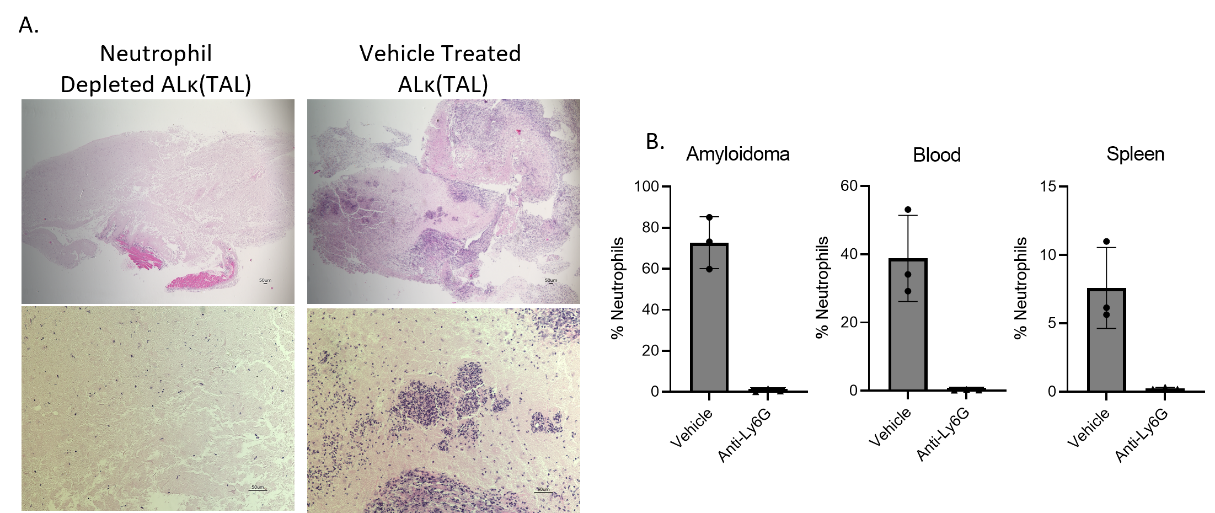


**Supplemental Figure 3 Evaluation of neutrophil depletion efficacy.** Mice were treated twice prior to amyloidoma implantation with either 250μg of Anti-Ly6G (1A8) antibody or vehicle intraperitoneally. 1 day post amyloid implantation tissue was harvested and analyzed histologically (A) or by flow cytometry (B). Systemic neutrophil depletion was evaluated in amyloid tissues, blood, and spleen by flow cytometry (B). Data is representative of 1 experiment with 5 animals per group (2 histology, 3 flow cytometry).
